# Supplementary material for: Intercellular communication between artificial cells by allosteric amplification of a molecular signal
Source: Nat Commun. 2020 Apr 3;11:1652. doi: 10.1038/s41467-020-15482-8 (PMC7125153; doi:10.1038/s41467-020-15482-8)
Supplement: Supplementary file 1 — Supplementary Information [file 41467_2020_15482_MOESM1_ESM.pdf]

Supplementary Information

Intercellular Communication between Artificial Cells by Allosteric Amplification of a Molecular Signal

Buddingh' et al.

Supplementary Figures

Supplementary Figure 1. Identifying the bottleneck in the Response Cascade

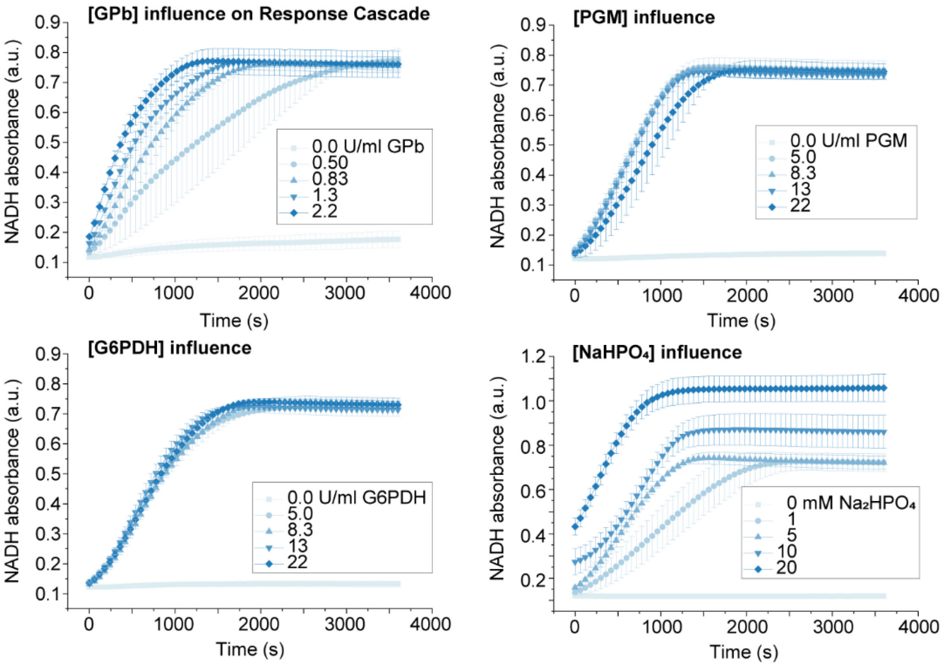

To optimize the Response Cascade, various concentrations of each enzyme as well as sodium phosphate were screened for their effect on the NADH output. In each of the four graphs the concentration of one of the enzymes or sodium phosphate was varied with all other conditions being equal.

The results demonstrated that both PGM and G6PDH were present in excess at all concentrations tested. The concentrations of GPb and Na_2HPO_4, on the other hand, were rate-limiting under these conditions. This is optimal for our purposes, as GPb is the signalling hub of the Response Cascade where amplification takes place. Therefore, the GPb-catalyzed conversion of glycogen into glucose-6-phosphate should be rate-limiting with all other enzymes present in excess to prevent the accumulation of intermediate metabolites that may provoke product inhibition. Furthermore, if one of the enzymes was missing or phosphate was not added, no output was generated, demonstrating the essential contribution of all components to the generation of NADH as output. All samples contained 1.0 mM AMP to activate the Response Cascade. Experiments were performed in duplicate and the error bars represent the SD.

Supplementary Figure 2. NADH output for a range of NAD^+ concentrations

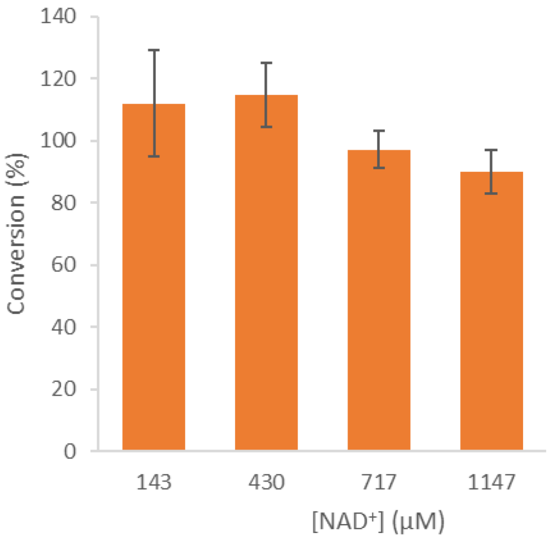

The optimized Response Cascade could convert NAD^+ fully into NADH, at concentrations of up to at least 1.1 mM NAD^+. Final conversions were calculated from the NADH absorbance at equilibrium. As can be seen in **Fig. 2c**, at higher NAD^+ concentrations the output increases even further, but did not reach equilibrium within the time frame of this experiment; hence, the conversion was not calculated here. Experiments were performed in triplicate and the error bars depict the SD.

Supplementary Figure 3. Balancing GPb and phosphate concentrations: speed vs. background activity

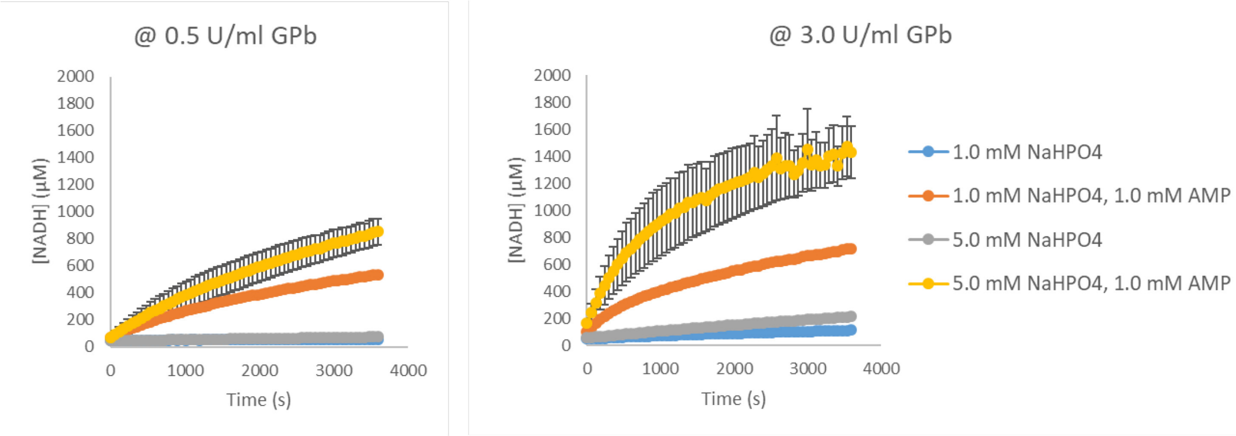

Comparison of the NADH output in presence of AMP vs. in its absence at different concentrations of Na_2HPO_4. Due to the low levels of constitutively active GPa that are present in the commercial GPb preparations, some background activity is always observed. At high concentrations of GPb (and therefore, GPa) and Na_2HPO_4, these are more pronounced than at lower concentrations. However, high concentrations of GPb and Na_2HPO_4 do increase both the speed and the output of the Response Cascade, respectively. The experiment was performed in duplicate and the error bars represent the standard deviation.

Supplementary Figure 4. Bulk test of Response Cascade for Figure 2h,i.

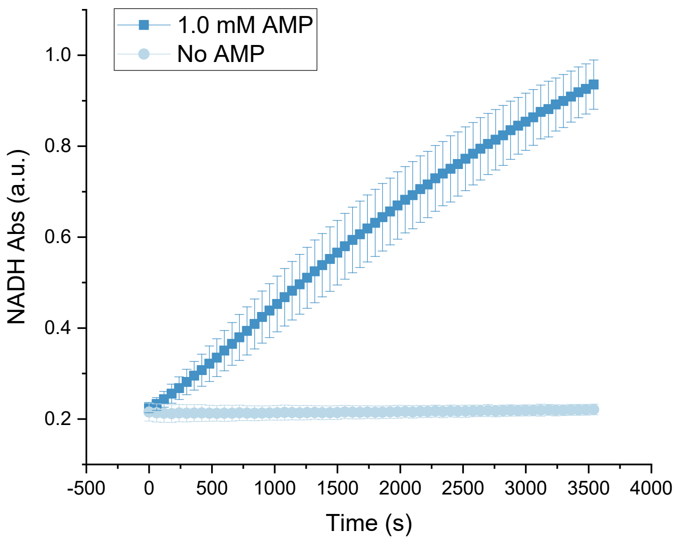

The function of the Response Cascade was tested in bulk reactions, using 1.0 mM Na_2HPO_4. The experiment was performed in duplicate and the error bars represent the standard deviation.

Supplementary Figure 5. Bulk test of Response Cascade for Figure 3b–d.

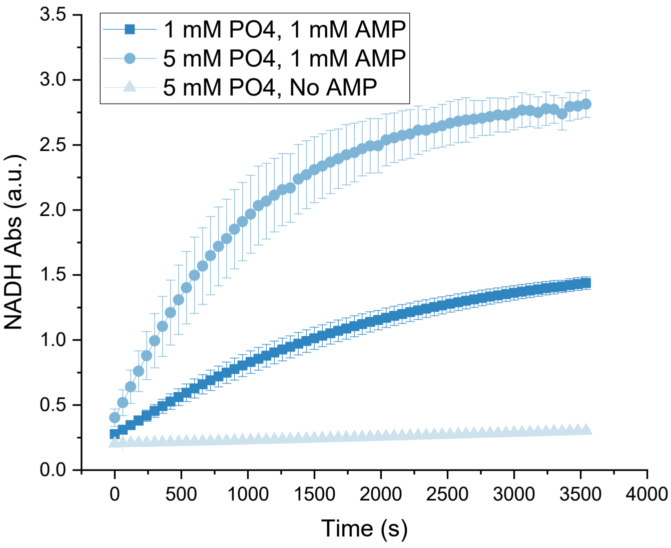

The function of the Response Cascade was tested in bulk reactions. The experiment was performed in duplicate and the error bars represent the standard deviation.

Supplementary Figure 6. Activation by AMP; individual Receiver GUVs

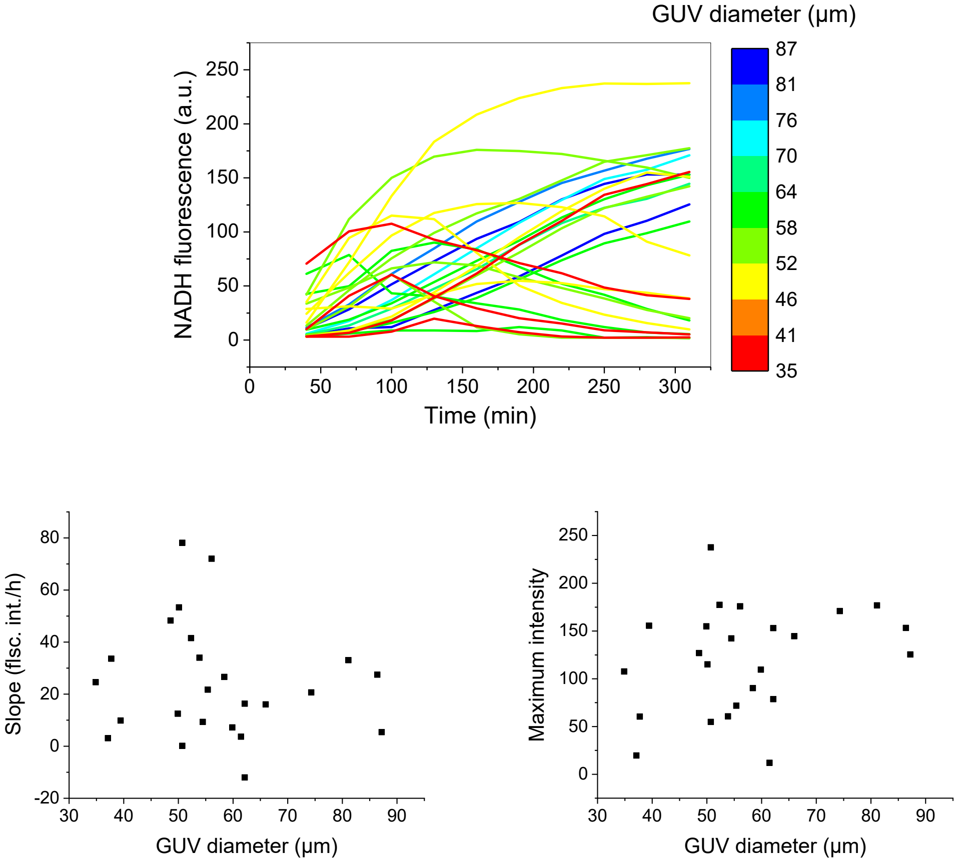

The upper panel displays the individual traces for the graph in **Figure 3a**. Each trace represents an individual Receiver GUV, activated by 1 mM AMP. The colour mapping relates the individual traces to the diameter of the corresponding GUV. The lower panels display the rate of activation of the Receivers (the initial increase in fluorescence intensity; left panel) and their amplitude (the maximum NADH fluorescence intensity; right panel). These data show that the observed heterogeneity in the activation of individual Receivers is not clearly related to their size. See the Supplementary Note for further discussion. n_{GUVs} = 24.

Supplementary Figure 7. Direct comparison of the activation of Receiver GUVs at different distances from an AMP or Na_2HPO_4 point source

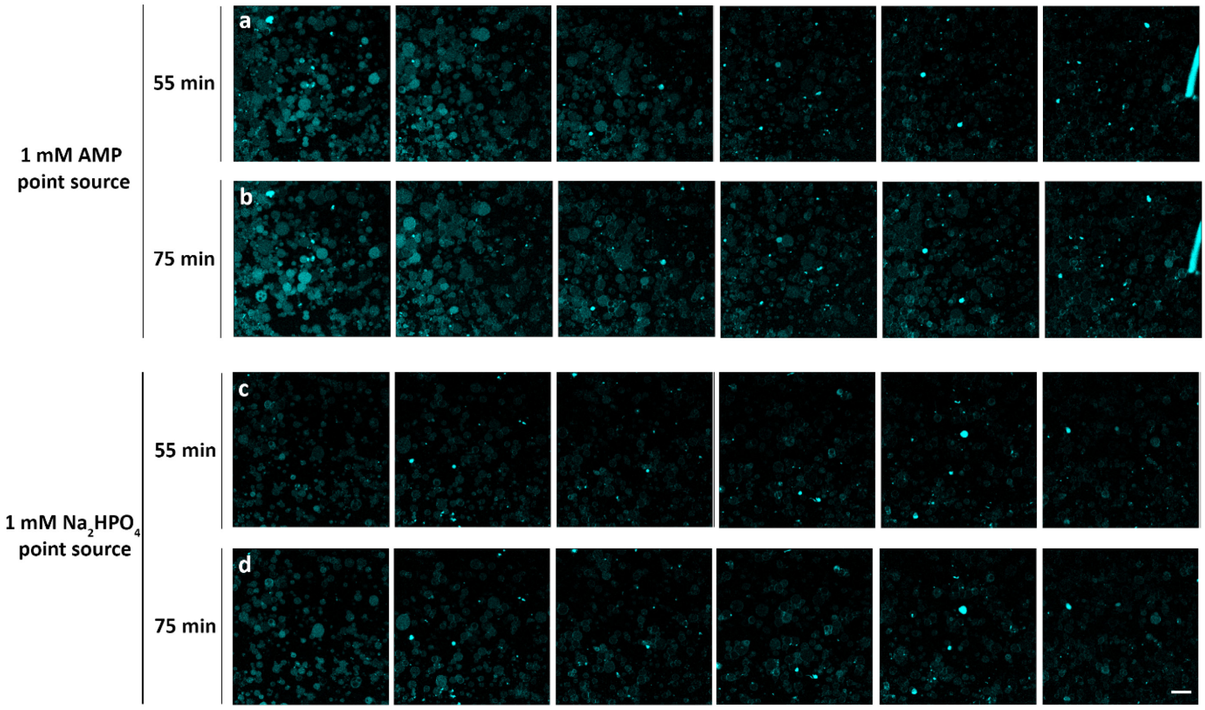

CLSM micrographs before GUV tracking and analysis as displayed in **Fig. 3b–d**. Each row displays micrographs of the NADH fluorescence at different distances from either an AMP (panel a,b) or Na_2HPO_4 (panel c,d) point source. AMP or Na_2HPO_4 was added to the left of the first micrograph along the row; the further right along the row, the farther away from the point source the Receiver GUVs are. Two time points are displayed; either 55 or 75 min after addition of AMP or Na_2HPO_4. Scale bar represents 50 µm.

Supplementary Figure 8. Direct comparison of the activation of Receiver GUVs at different distances from an AMP or Na_2HPO_4 point source

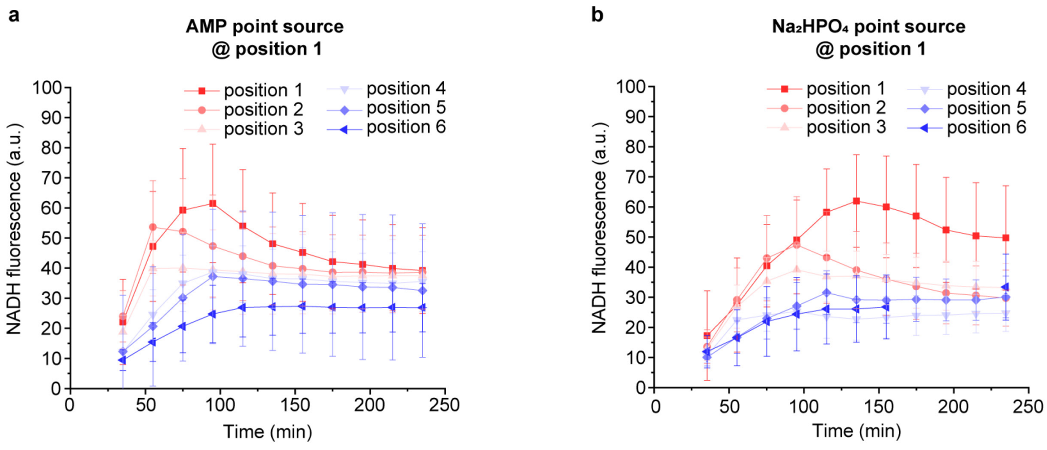

These graphs display the distance-dependent activation of Receivers when **a**, 1 mM AMP was added as a point source, with 1 mM Na_2HPO_4 homogeneously spread throughout the sample, or **b**, 1 mM Na_2HPO_4 was added as a point source, with 1 mM AMP homogeneously spread throughout the sample. The point sources were located at position 1; the other positions are progressively further removed from the point source, with equal spacing between positions. The data were extracted from the images in **Suppl. Fig. 7** and related images using Fiji's particle analysis plug-in. **Figure 3d** displays the same data, yet grouped differently to directly compare the allosteric activation and substrate activation at four selected positions. Error bars depict the SD from analyzing 40–200 GUVs per position.

Supplementary Figure 9. Washing of GUVs removes unencapsulated proteins

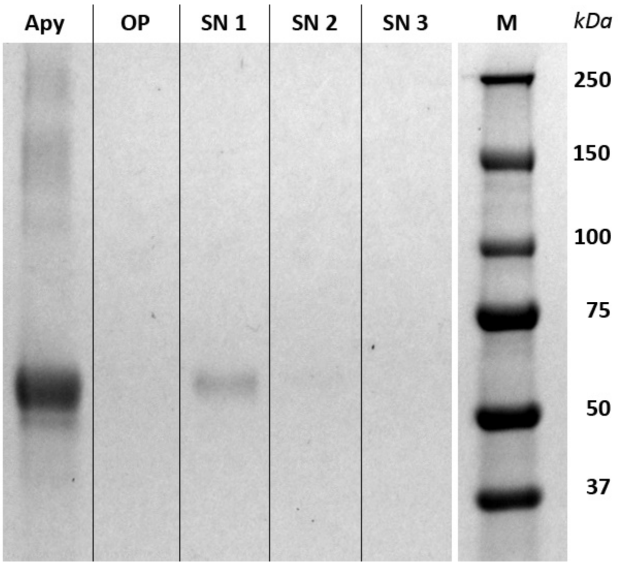

Washing apyrase-loaded GUVs according to the centrifugation protocol described in the Methods section removed any unencapsulated components from the external solution. Apy = apyrase solution, OP = fresh external solution (=outer phase), SN = supernatant, M = protein marker, the molecular weights of which are indicated.

Before the first washing step apyrase was present in the supernatant (SN 1); after the first washing step it was still present at low concentrations (SN 2); the second washing step removed all remaining apyrase that was not encapsulated in the GUVs. The purified GUVs were used for subsequent experiments. The reported molecular weight of apyrase is 49 kDa.[1]

Supplementary Figure 10. ATP and ADP conversion into AMP activates the Response Cascade

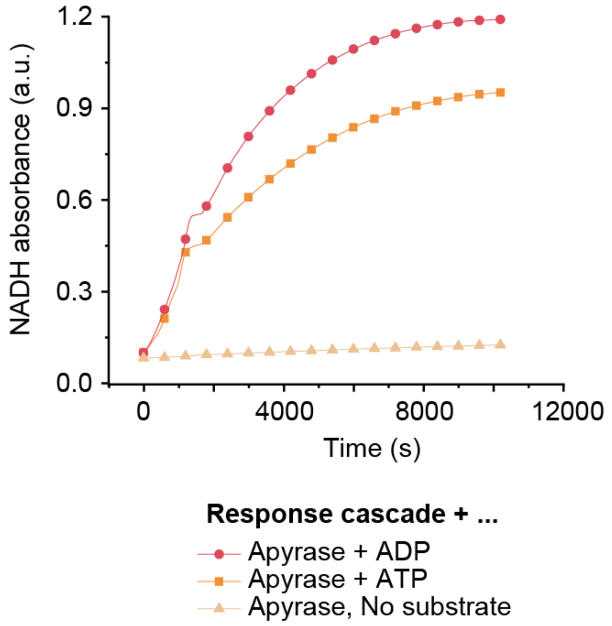

Bulk reactions showed that apyrase can convert ATP and ADP into AMP, which in turn activated the Response Cascade. In absence of either substrate, AMP was not generated and the Response Cascade did not activate.

Supplementary Figure 11. ATP by itself does not activate the Response Cascade

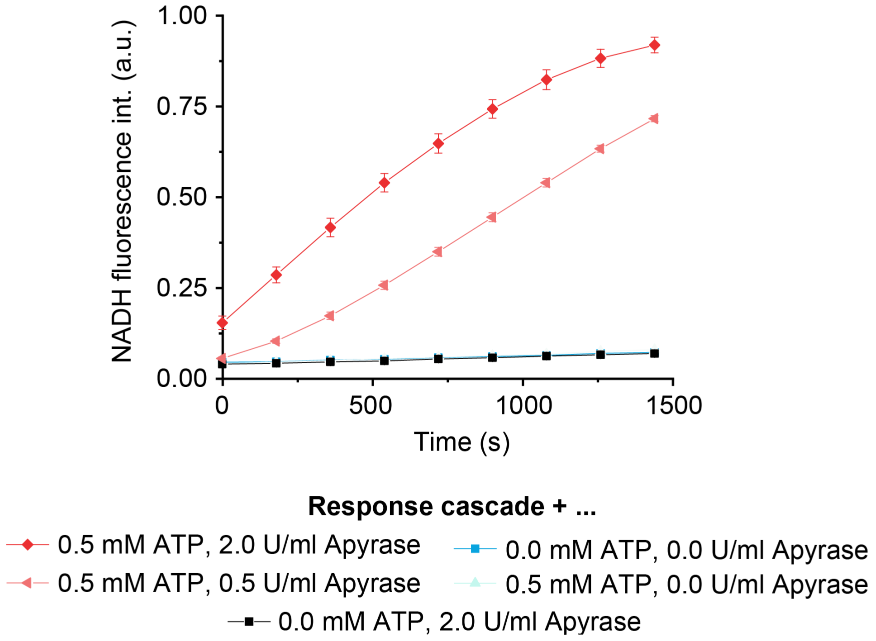

Bulk reactions showed that ATP could not activate the Response Cascade when Apyrase was absent. Thus, non-enzymatic hydrolysis of ATP plays a negligible role in activation of the Receivers. Similarly, Apyrase could not activate the Response Cascade in absence of ATP. Error bars depict the SD.

Supplementary Figure 12. Activation of Receivers by Senders; individual Receiver GUVs

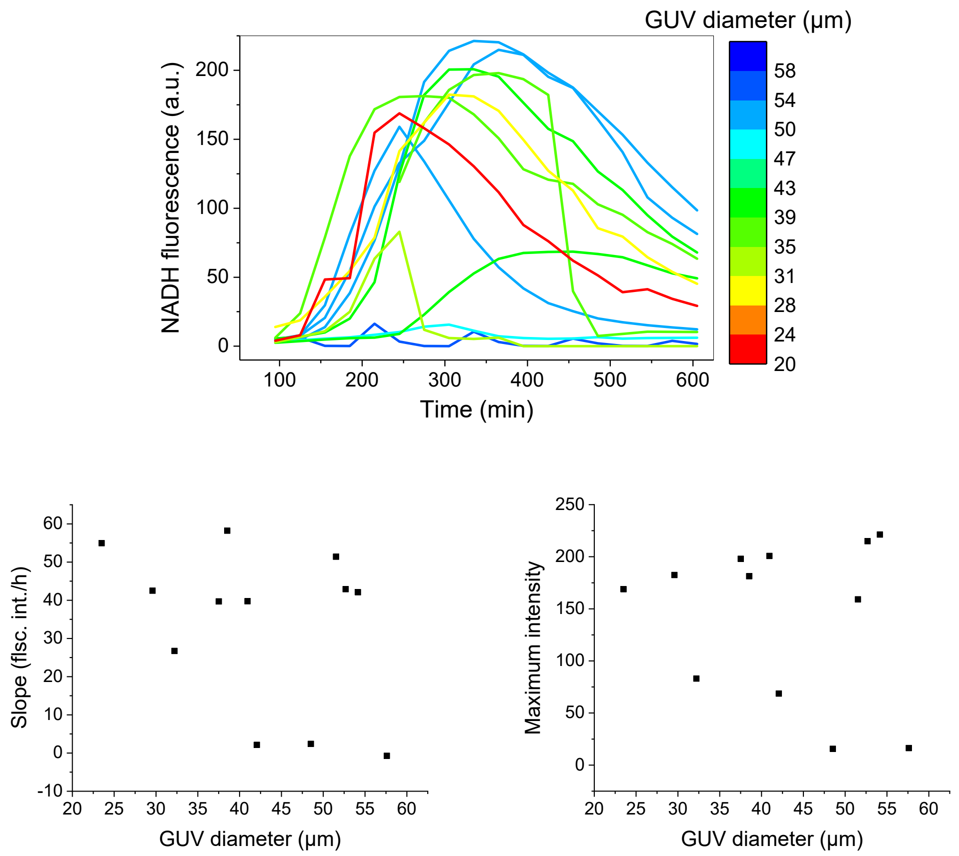

The upper panel displays the individual traces for the graph in **Figure 6d**. Each trace represents an individual Receiver GUV, activated by the surrounding Sender GUVs. The colour mapping relates the individual traces to the diameter of the corresponding Receiver GUV. The lower panels display the rate of activation of the Receivers (the initial increase in fluorescence intensity; left panel) and their amplitude (the maximum NADH fluorescence intensity; right panel). These data show that the observed heterogeneity in the activation of individual Receivers is not clearly related to their size. See the Supplementary Note for further discussion. n_{GUVs} = 12.

Supplementary Figure 13. αHL insertion into GUV membranes

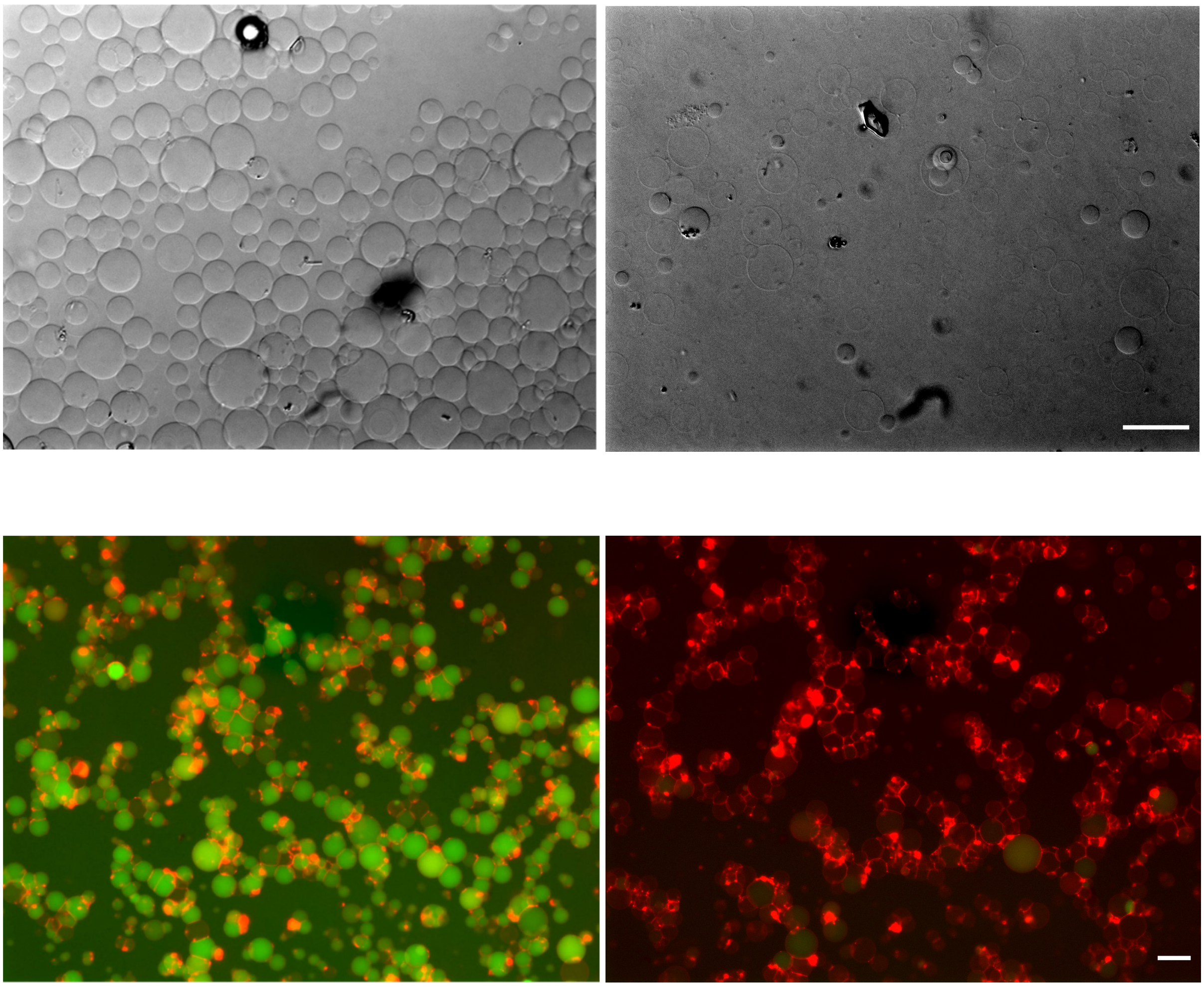

αHL self-inserted into GUV membranes and permeabilized most GUVs. The kinetics of protein insertion and the release of low-molecular weight contents were heterogeneous amongst the GUV population.

Top row: brightfield micrographs of GUVs; left) before αHL addition, right) after αHL insertion. Before αHL insertion, the sucrose/sorbitol gradient created a difference in refractive index between the lumen of the GUVs and the external solution. This was lost upon equilibration of the sucrose/sorbitol gradient through αHL pores. The GUV membrane composition was 35/35/30 (mol/mol) DOPC/POPC/cholesterol + 1.0% DSPE-PEG2000-biotin. [αHL] = 83 μg ml^{-1}. Scale bar represents 50 μm.

Bottom row: calcein release from GUVs upon αHL insertion; left) right after αHL addition, right) t = 174 min. GUV membrane composition was 70/30 DOPC/cholesterol + 0.06% DOPE-LRB (red). Calcein (1.0 mM, green) was encapsulated in the GUVs during their assembly. [αHL] = 33 μg ml^{-1}. Scale bar represents 50 μm.

Supplementary Figure 14. αHL insertion into GUV membranes (2)

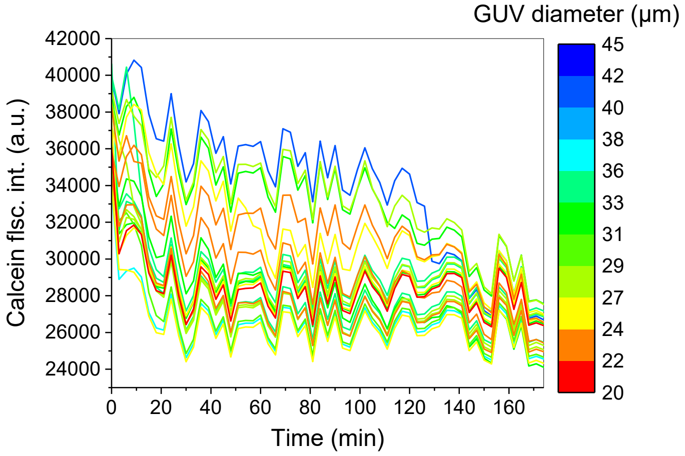

Calcein-release profiles for individual GUVs. Each trace represents an individual, calcein-filled GUV after addition of αHL. GUVs were randomly selected from **Supplementary Figure 13**, bottom row. (n_{GUVs} = 20). The colour mapping relates the individual traces to the diameter of the corresponding GUV. These data show the heterogeneous calcein-release profiles of individual GUVs in response to incubation with αHL. The size of the GUVs does not appear to play a role in the kinetics of αHL insertion and calcein-release. See the Supplementary Note for further discussion.

Supplementary Figure 15. Supplementary images for Figure 3a

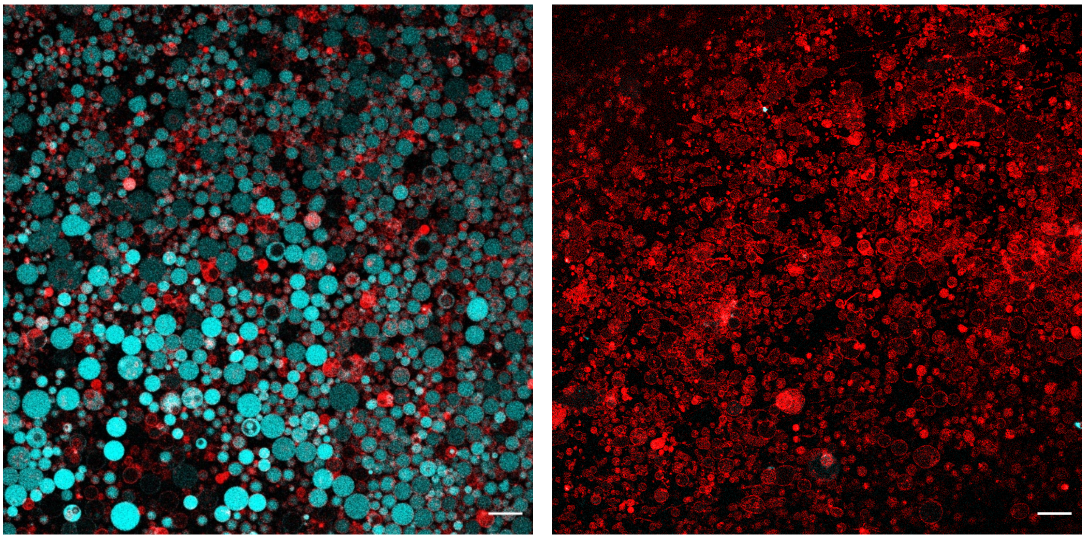

Receivers in presence of left) 5.0 mM AMP, right) no AMP. Overlay of NADH (cyan) and Receiver GUVs (red). Scale bars represent 50 µm.

Supplementary Figure 16. Supplementary images for Figure 4b–g

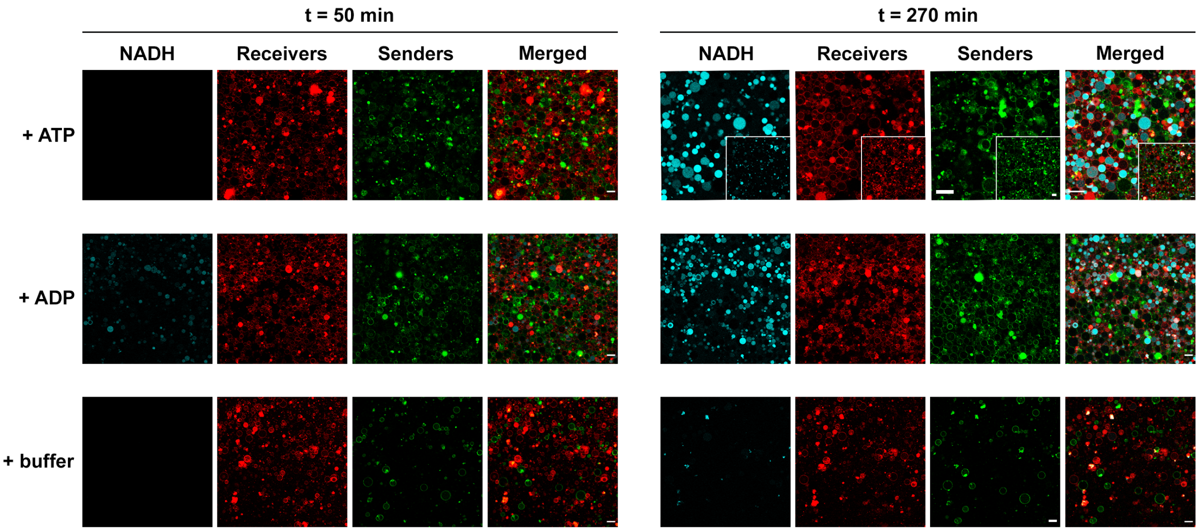

Individual fluorophore channels for the images in **Fig. 4b–g**. NADH fluorescence is pseudo-coloured cyan; Receivers incorporate the fluorescent lipid DOPE-(Lissamine Rhodamine B) (red); Sender GUVs incorporate DOPE-carboxyfluorescein (green). Scale bars represent 50 µm.

Supplementary Figure 17. Supplementary images for Figure 5

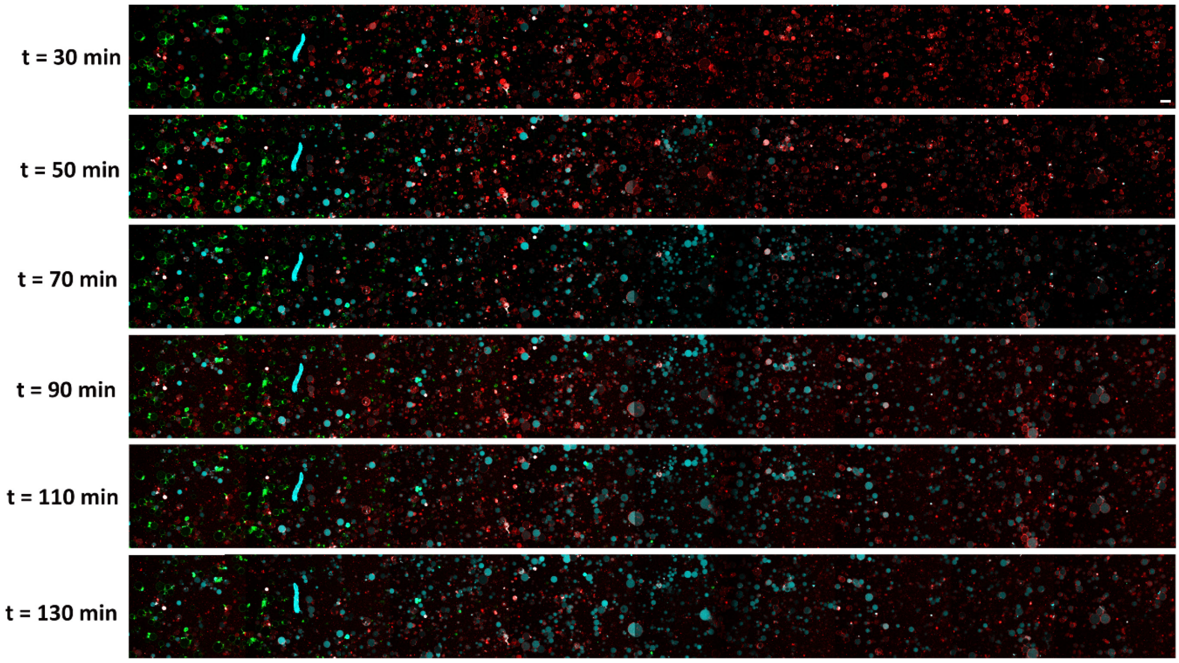

More time points from the experiment in **Fig. 5a**. Overlay of NADH (cyan), Sender GUVs (green), Receiver GUVs (red). Scale bar represents 50 μm.

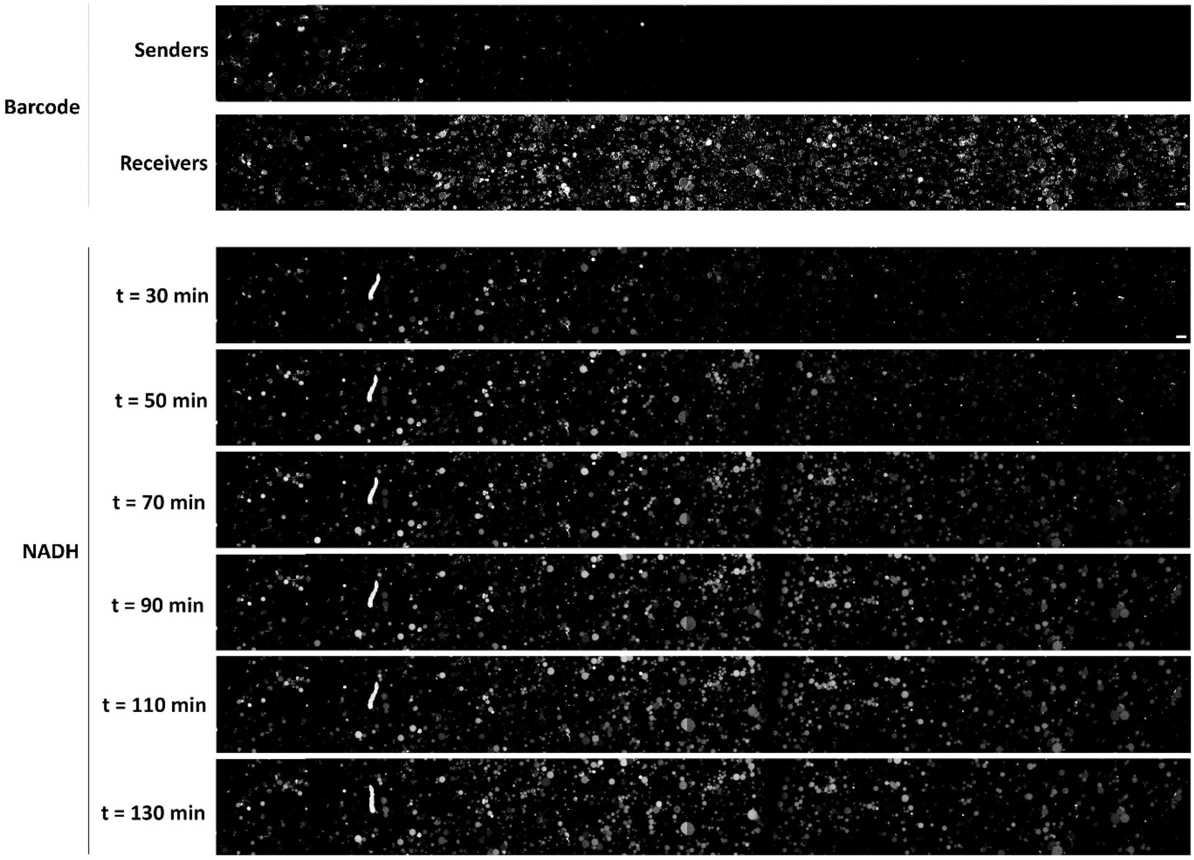

Individual fluorophore channels for the image above. Barcodes for Senders and Receivers are based on their respective fluorescent lipids, DOPE-(Lissamine Rhodamine B) and DOPE-carboxyfluorescein, respectively. The bottom rows show the NADH fluorescence at various time points without the fluorescent GUV membranes. Scale bar represents 50 µm.

Supplementary Figure 18. Supplementary images for Figure 5 (2)

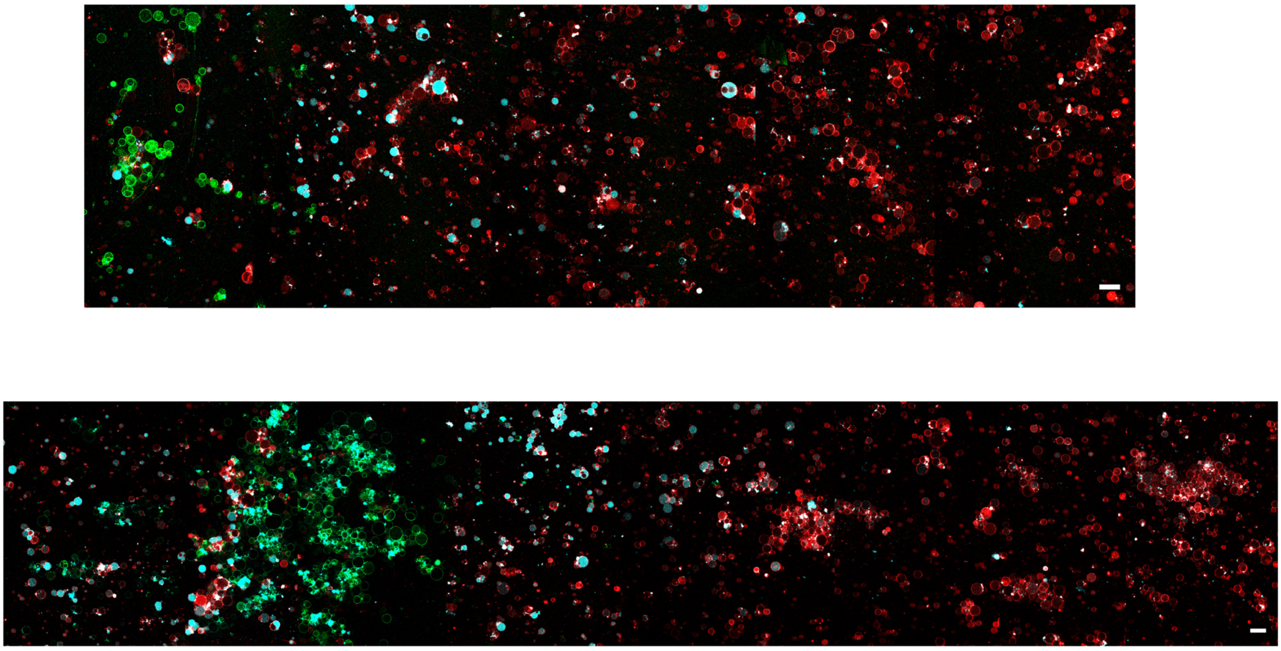

Experimental replications of the experiment in **Fig. 5a**, demonstrating the reproducibility of the observed signaling front. t = 50 min for both micrographs. Scale bars are not included because the images did not completely overlap. Overlay of NADH (cyan), Sender GUVs (green), Receiver GUVs (red). Scale bars represent 50 µm.

Supplementary Figure 19. Supplementary images for Figure 6b

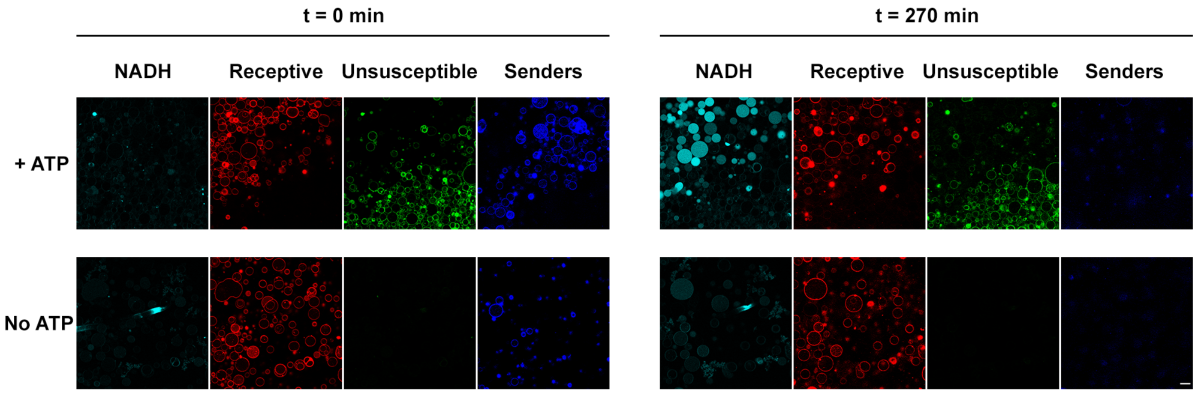

Individual fluorophore channels for the images in **Fig. 6b**. NADH fluorescence is pseudo-coloured cyan; Receptive Receivers incorporate the fluorescent lipid DOPE-(Lissamine Rhodamine B) (red); Unsusceptible Receivers incorporate DOPE-carboxyfluorescein (green); Sender GUVs incorporate DOPE-cyanine 5 (blue). Scale bar represents 30 µm.

Supplementary Figure 20. Supplementary images for Figure 6b (2)

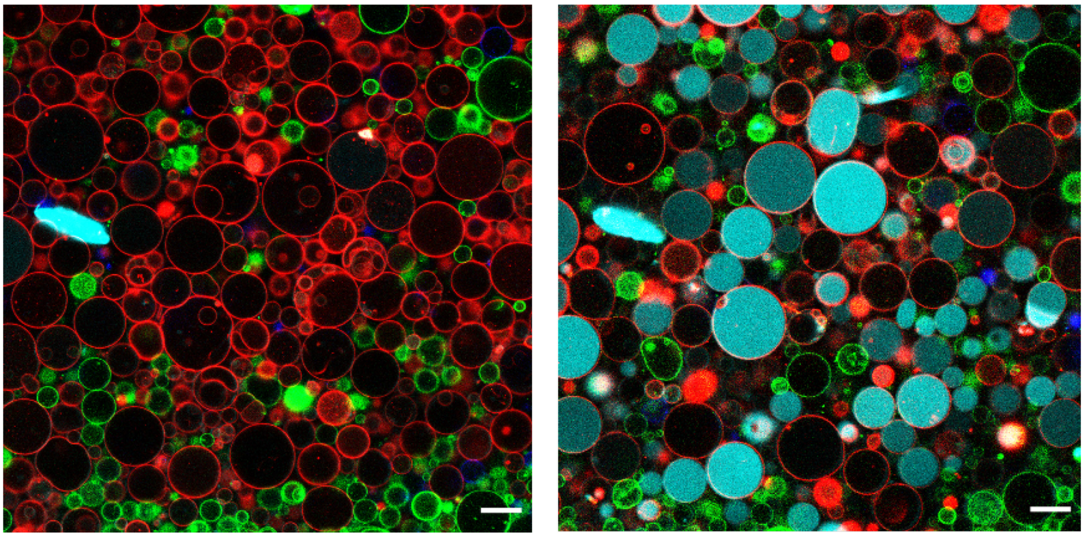

Replications of the experiment in **Fig. 6b**, demonstrating the reproducibility of the observed selective signalling. Senders, receptive and unsusceptible Receivers in presence of 2.6 mM ATP. Left: t = 30 min; right: t = 270 min. Overlay of NADH (cyan), Sender GUVs (blue), receptive Receivers (red), unsusceptible Receivers (green). Scale bars represent 30 µm.

Supplementary Figure 21. NADH calibration curve

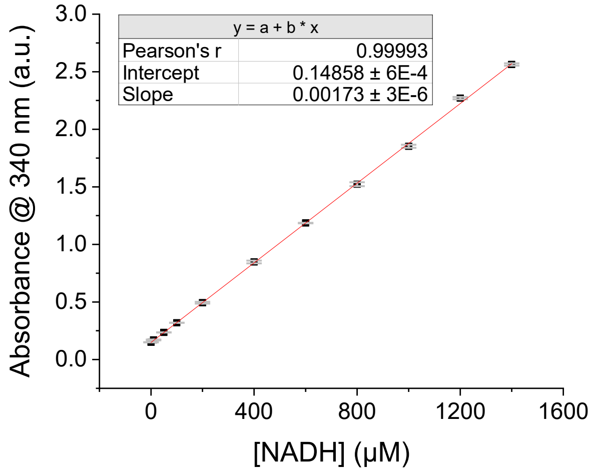

Calibration curve for the bulk reactions. All NADH concentrations were measured in the appropriate buffer and measurements were performed in triplicate. Error bars represent the SD.

Supplementary Figure 22. Design of the channel device

Top view:

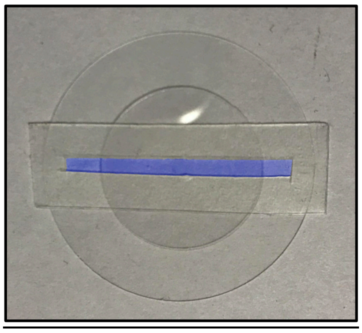

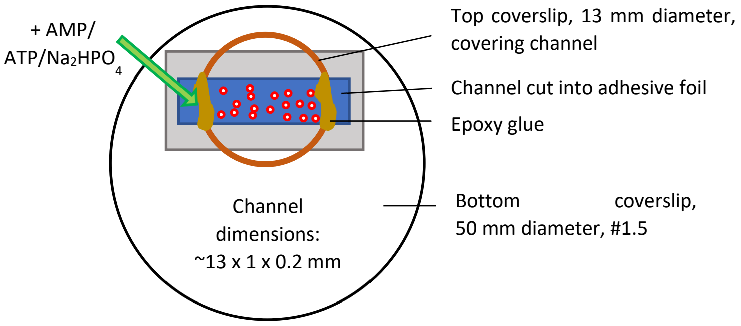

Side view

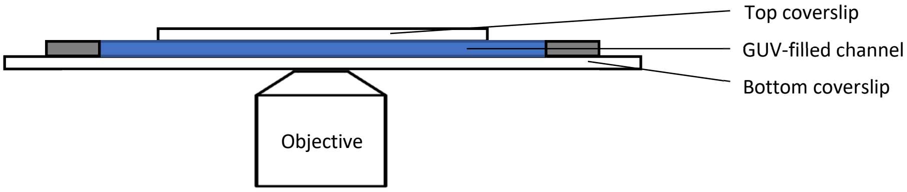

Imaging procedure:

1. Load channel with outer phase solution
2. Add GUVs to A
3. Seal A
4. Add AMP/ATP/Na_2HPO_4 to B
5. Seal B

Supplementary Notes

Supplementary Note 1. Optimization of the enzymatic reactions

Sender

Apyrase was used to generate the AMP since it has a high turnover and, therefore, can produce AMP efficiently. It hydrolyzes the γ-phosphate of ATP, and the ß-phosphate of ADP to generate AMP and two equivalents of orthophosphate. The enzyme from *Solanum tuberosum* has two isozymes: one with an ATPase/ADPase ratio of ~10/1 and one with similar ATPase and ADPase activities. Since the ADP is a non-active intermediate whose build-up would be undesirable, we opted for the isozyme with an ATPase/ADPase ratio of ~1. It requires Ca^{2+} for optimal function, which is why this was included in the solutions.[1,2]

Since ATP is an important metabolite in the energy metabolism of cells and known to inhibit GPb as part of a negative feedback loop, we investigated its influence on the Response Cascade. Low millimolar concentrations of ATP did not inhibit the activation, but at >5 mM ATP inhibition was observed. Therefore, ATP concentrations below 5 mM were used throughout this study.

Response Cascade

The Response Cascade was built using three important enzymes from the glycogen metabolism and the pentose phosphate pathway. Glycogen phosphorylase was selected because its unphosphorylated form, glycogen phosphorylase b, can be allosterically activated by AMP, and would as such provide an inducible enzymatic reaction that leads to high levels of signal amplification. It hydrolyzes a terminal α-1,4-glycosidic bond using orthophosphate and produces glucose-1-phosphate and a shorter glycogen chain. The phosphorylated isoform of GP, called GPa, is constitutively active and insensitive to AMP. The presence of low levels of GPa in the commercial GPb preparation accounts for the weak NADH output that can be observed in the absence of AMP. Activation of GPb by addition of AMP, however, induces a far stronger NADH output, clearly providing a strong on/off *modus operandi* for the Response Cascade.

To generate a physiologically relevant output from the Response Cascade, we selected PGM and G6PDH to convert the product of GPb, glucose-1-phosphate (G1P), into NADH. Additionally, this provided a convenient read-out for the activation of GPb, because NADH is fluorescent with an excitation maximum at 340 nm and an emission maximum around 460 nm. PGM needs divalent cations for its activity (especially Mg^{2+}) as well as α-D-glucose-1,6-bisphosphate (as a phosphate donor for the serine residue in the active site). The equilibrium between G1P and G6P that is catalyzed by PGM has a ΔG of -7.5 kJ mol^{-1},[3] therefore favoring the conversion into G6P.

The concentrations of all three enzymes were optimized as shown in **Suppl. Figure 1**; furthermore, the concentrations of the other components were optimized as well. Subsequently, the kinetics and amplitude of the Response Cascade could be tuned as outlined in the main text. Due to the presence of a small amount of constitutively active GPa in the commercial GPb preparation, some background activity in the absence of AMP is always observed. Therefore, a trade-off has to be made: if a fast

reaction (to get an NADH burst) is required, high concentrations of GPb (3.0 U ml^{-1} vs. 0.5 U ml^{-1}) and Na_2HPO_4 are beneficial. If, however, the background reaction should be suppressed (for instance because of the time needed for AMP to diffuse to the Receiver GUVs), low concentrations of GPb and Na_2HPO_4 are preferable (**Suppl. Figure 3**).

Supplementary Note 2. Activating the Response Cascade in GUVs

To generate the Receivers, 200 mM sorbitol was used in the outer phase solution instead of the widely used glucose, as glucose was shown to have an inhibiting effect on the Response Cascade at > 50 mM; most likely this is caused by a negative product feedback loop in the glucose metabolism.

Since AMP is anionic, it cannot readily permeate phospholipid membranes. Therefore, α-hemolysin was added to the GUVs to allow the influx and efflux of small metabolites like AMP through the 1.4 nm pores.[4] However, since only macromolecules like enzymes and glycogen could be retained inside the GUVs, all other metabolites and ions, like NAD^+ and $CaCl_2$, were added to the external solution as well to prevent drainage of the Response Cascade from the Receivers.

Supplementary Note 3. Artificial cell heterogeneity

As discussed in the main text, throughout our experiments we observed a marked heterogeneity in the response of the Receivers to AMP (**Fig. 3a**, **4a**, **6d**). Although the population of Receivers as a whole successfully detected the presence of AMP, interesting differences in lag time, kinetics and yield of NADH production were observed. The many variables involved in the activation of the Receivers confound the establishment of a clear causal relationship between input parameters – such as GUV size, permeability and loading – and the response of individual Receivers. A clear correlation with the size of the GUVs was not observed (**Supplementary Figures 6**, **12**). Moreover, the size of the GUVs and the enzyme concentrations used here implicate that each GUV contained 10^6–10^7 enzymes (see table below). Therefore, stochastic fluctuations in solute partitioning during GUV formation are not considered to be likely causes of the observed variability in the response of the artificial cells. Other non-stochastic effects, however, may play an important role in solute distribution.[5]

Average GUV diameter = 50 µm		Average GUV volume = 65 pL	
**Enzyme**	**Activity (U ml^{-1})**	**Concentration (µg ml^{-1})**	**Concentration (nM)**
GPb	0.5	19	195
PGM	5.0	26	442
G6PDH	5.0	13	120

Most likely, differential permeability of the GUVs has a more significant influence on the activation kinetics and NADH output of individual GUVs. As αHL inserts into the phospholipid bilayers spontaneously, controlling the degree of insertion per vesicle is difficult, which causes divergent in- and efflux rates of small polar molecules (**Supplementary Figure 14**). Moreover, several studies have pointed out the existence of subpopulations of GUVs with significantly more permeable membranes – even in the absence of membrane pores.[6,7] Finally, although GUVs produced by the droplet transfer

technique are generally unilamellar, a fraction of vesicles can be expected to be multilamellar. This would also have strong influence on the permeability of an individual GUV.

A more detailed analysis of the different factors that govern the heterogeneity of the response to AMP would establish the important functional consequences of artificial cell heterogeneity, yet requires a full quantification of all relevant concentrations, permeability coefficients, etc. for individual vesicles. Such further quantitative studies – although outside the scope of the current study – will likely provide important insights for the implementation of cell-like communication networks.

Supplementary Note 4. List of abbreviations

ADP	adenosine 5'-diphosphate
αHL	alpha-haemolysin
AMP	adenosine 5'-monophosphate
ATP	adenosine 5'-triphosphate
BSA	bovine serum albumin
CLSM	confocal laser scanning microscopy
DNA	deoxyribonucleic acid
G1P	α-D-glucose-1-phosphate
G6P	D-glucose-6-phosphate
G6PDH	D-glucose-6-phosphate dehydrogenase
GBP	α-D-glucose-1,6-bisphosphate
GPa	glycogen phosphorylase a
GPb	glycogen phosphorylase b
GUV	giant unilamellar vesicle
HBS	HEPES-buffered saline
λ_{ex}	excitation wavelength
λ_{em}	emission wavelength
mRNA	messenger ribonucleic acid
MWCO	molecular weight cut-off
NAD^+	ß-nicotinamide adenine dinucleotide (oxidized)
NADH	ß-nicotinamide adenine dinucleotide (reduced)
PGM	phosphoglucomutase
SD	standard deviation

Phospholipids

DOPC	1,2-dioleoyl-*sn*-glycero-3-phosphocholine
DOPE-CF	1,2-dioleoyl-*sn*-glycero-3-phosphoethanolamine-*N*-(carboxyfluorescein)
DOPE-Cy5	1,2-dioleoyl-*sn*-glycero-3-phosphoethanolamine-*N*-(cyanine 5)
DOPE-LRB	1,2-dioleoyl-*sn*-glycero-3-phosphoethanolamine-*N*-(lissamine rhodamine B sulfonyl)
DSPE-PEG-biotin	1,2-distearoyl-*sn*-glycero-3-phosphoethanolamine-*N*-[biotinyl(polyethylene glycol)-2000]
POPC	1-palmitoyl-2-oleoyl-glycero-3-phosphocholine

Supplementary Methods

Concentrations of key reagents for each experiment.

**Figure 2a, GPb series**	
Solution	
HBS	1X
Sorbitol	200 mM
$MgCl_2$	2.25 mM
$CaCl_2$	5.8 mM
GBP	8 μM
NAD^+	430 μM
Glycogen	2.0 mg/ml
GPb	As indicated
PGM	5.0 U/ml
G6PDH	5.0 U/ml
K_2HPO_4	1.1 mM
AMP	1.0 mM

**Figure 2c, NAD^+ series**	
Solution	
HBS	1X
Sorbitol	200 mM
$MgCl_2$	2.25 mM
$CaCl_2$	5.8 mM
GBP	8 μM
NAD^+	As indicated
Glycogen	2.0 mg/ml
GPb	0.50 U/ml
PGM	5.0 U/ml
G6PDH	5.0 U/ml
K_2HPO_4	5.0 mM
AMP	1.0 mM

**Figure 2d,-g, AMP vs. Na_2HPO_4**	
Solution	
HBS	1X
Sorbitol	200 mM
$MgCl_2$	2.25 mM
$CaCl_2$	5.8 mM
GBP	8 μM
NAD^+	2.0 mM
Glycogen	2.0 mg/ml
GPb	0.50 U/ml
PGM	5.0 U/ml
G6PDH	5.0 U/ml
Na_2HPO_4	As indicated
AMP	As indicated

**Figure 3a, AMP activation of Receivers (graph + isolated GUVs)**			
**Receivers**		**Outer phase**	
DOPC/Chol	70/30	HBS	1X
DSPE-PEG	1%	Sorbitol	200 mM
HBS	1X	$MgCl_2$	2.25 mM
Sucrose	200 mM	$CaCl_2$	5.8 mM
$MgCl_2$	2.25 mM	GBP	8 μM
$CaCl_2$	5.8 mM	NAD^+	430 μM
GBP	8 μM	αHL	20 μg/ml
NAD^+	430 μM	K_2HPO_4	1.1 mM
Glycogen	2.0 mg/ml	AMP	As indicated
GPb	0.50 U/ml		
PGM	5.0 U/ml		
G6PDH	5.0 U/ml		

**Figure 3a, AMP activation of Receivers (overview images + movie S4)**			
**Receivers**		**Outer phase**	
DOPC/POPC/Chol	35/35/30	HBS	1X
DSPE-PEG	1%	Sorbitol	200 mM
HBS	1X	$MgCl_2$	2.25 mM
Sucrose	200 mM	$CaCl_2$	5.8 mM
$MgCl_2$	2.25 mM	GBP	8 μM
$CaCl_2$	5.8 mM	NAD^+	2.0 mM
GBP	8 μM	αHL	75 μg/ml
NAD^+	2.0 mM	Na_2HPO_4	1.0 mM
Glycogen	2.0 mg/ml	AMP	As indicated
GPb	1.0 U/ml		
PGM	5.0 U/ml		
G6PDH	5.0 U/ml		
Purified GUVs			

**Suppl. Fig. 1, Response Cascade bottleneck**	
Solution	
HBS	1X
Sorbitol	200 mM
$MgCl_2$	2.25 mM
$CaCl_2$	5.8 mM
GBP	8 μM
NAD^+	430 μM
Glycogen	2.0 mg/ml
GPb	As indicated
PGM	As indicated
G6PDH	As indicated
Na_2HPO_4	As indicated
AMP	1.0 mM

**Suppl. Fig. 3, GPb and PO_4 tuning**	
Solution	
HBS	1X
Sorbitol	200 mM
$MgCl_2$	2.25 mM
$CaCl_2$	5.8 mM
GBP	8 μM
NAD^+	2.0 mM
Glycogen	2.0 mg/ml
GPb	As indicated
PGM	5.0 U/ml
G6PDH	5.0 U/ml
Na_2HPO_4	As indicated
AMP	As indicated

**Figure 2h,i, Kymograms of Response Cascade**	
Solution	
HBS	1X
Sorbitol	200 mM
$MgCl_2$	2.25 mM
$CaCl_2$	5.8 mM
GBP	8 μM
NAD^+	2.0 mM
Glycogen	2.0 mg/ml
GPb	0.50 U/ml
PGM	5.0 U/ml
G6PDH	5.0 U/ml
Na_2HPO_4	As indicated
AMP	As indicated

Figure 3b,c,d, Kymograms of Receivers			
**Receivers**		**Outer phase**	
DOPC/POPC/Chol	35/35/30	HBS	1X
DSPE-PEG	1%	Sorbitol	200 mM
HBS	1X	$MgCl_2$	2.25 mM
Sucrose	200 mM	$CaCl_2$	5.8 mM
$MgCl_2$	2.25 mM	GBP	8 µM
$CaCl_2$	5.8 mM	NAD^+	2.0 mM
GBP	8 µM	αHL	10 µg/ml
NAD^+	2.0 mM	Na_2HPO_4	As indicated
Glycogen	2.0 mg/ml	AMP	As indicated
GPb	2.5 U/ml		
PGM	5.0 U/ml		
G6PDH	5.0 U/ml		
Purified GUVs			

Suppl. Fig. 10, AMP formation by apyrase	
Solution	
HBS	1X
Sorbitol	200 mM
$MgCl_2$	2.25 mM
$CaCl_2$	5.8 mM
GBP	8 µM
NAD^+	2.0 mM
Glycogen	1.2 mg/ml
GPb	0.30 U/ml
PGM	3.0 U/ml
G6PDH	3.0 U/ml
Apyrase	15 U/ml
Na_2HPO_4	5.0 mM
ADP/ATP	As indicated

Figure 4, Activation of Receivers by Senders, AMP/ADP/ATP					
**Senders**		**Receivers**		**Outer phase**	
DOPC/POPC/Chol	35/35/30	DOPC/POPC/Chol	35/35/30	HBS	1X
DSPE-PEG	1%	DSPE-PEG	1%	Sorbitol	200 mM
HBS	1X	HBS	1X	$MgCl_2$	2.25 mM
Sucrose	200 mM	Sucrose	200 mM	$CaCl_2$	5.8 mM
$MgCl_2$	2.25 mM	$MgCl_2$	2.25 mM	GBP	8 µM
$CaCl_2$	5.8 mM	$CaCl_2$	5.8 mM	NAD^+	2.0 mM
GBP	8 µM	GBP	8 µM	αHL	150 µg/ml
NAD^+	2.0 mM	NAD^+	2.0 mM	Na_2HPO_4	5.0 mM
Apyrase	39 U/ml	Glycogen	2.0 mg/ml	AMP/ADP/ATP	As indicated
		GPb	0.50 U/ml		
		PGM	5.0 U/ml		
		G6PDH	5.0 U/ml		

Figure 6, Receptive vs. unsusceptible Receivers					
**Senders**		**Receivers**		**Outer phase**	
DOPC/POPC/Chol	35/35/30	DOPC/POPC/Chol	35/35/30	HBS	1X
DSPE-PEG	1%	DSPE-PEG	1%	Sorbitol	200 mM
HBS	1X	HBS	1X	$MgCl_2$	2.25 mM
Sucrose	200 mM	Sucrose	200 mM	$CaCl_2$	5.8 mM
$MgCl_2$	2.25 mM	$MgCl_2$	2.25 mM	GBP	8 µM
$CaCl_2$	5.8 mM	$CaCl_2$	5.8 mM	NAD^+	2.0 mM
GBP	8 µM	GBP	8 µM	αHL	50 µg/ml
NAD^+	2.0 mM	NAD^+	2.0 mM	Na_2HPO_4	5.0 mM
Apyrase	39 U/ml	Glycogen	2.0 mg/ml	ATP	As indicated
Purified GUVs		GPb	0 or 2.5 U/ml		
		PGM	5.0 U/ml		
		G6PDH	5.0 U/ml		

Figure 5, Signaling front					
**Senders**		**Receivers**		**Outer phase**	
DOPC/POPC/Chol	35/35/30	DOPC/POPC/Chol	35/35/30	HBS	1X
DSPE-PEG	1%	DSPE-PEG	1%	Sorbitol	200 mM
HBS	1X	HBS	1X	$MgCl_2$	2.25 mM
Sucrose	200 mM	Sucrose	200 mM	$CaCl_2$	5.8 mM
$MgCl_2$	2.25 mM	$MgCl_2$	2.25 mM	GBP	8 µM
$CaCl_2$	5.8 mM	$CaCl_2$	5.8 mM	NAD^+	2.0 mM
GBP	8 µM	GBP	8 µM	αHL	50 µg/ml
NAD^+	2.0 mM	NAD^+	2.0 mM	Na_2HPO_4	10 mM
Apyrase	77 U/ml	Glycogen	2.0 mg/ml	ATP	2.6 mM
Purified GUVs		GPb	5.0 U/ml		
		PGM	5.0 U/ml		
		G6PDH	5.0 U/ml		

Supplementary References

1. Kettlun, A. M. *et al.* Properties of two apyrases from Solanum tuberosum. *Phytochemistry* **21**, 551–558 (1982).
2. Molnar, J. & Lorand, L. Studies on apyrases. *Arch. Biochem. Biophys.* **93**, 353–363 (1961).
3. Berg, J. M., Tymoczko, T. L., Gatto Jr., G. J. & Stryer, L. *Biochemistry*. (W.H. Freeman & Co, 2015).
4. Song, L. *et al.* Structure of Staphylococcal alpha -Hemolysin, a Heptameric Transmembrane Pore. *Science* **274**, 1859–1865 (1996).
5. Altamura, E., Carrara, P., D'Angelo, F., Mavelli, F. & Stano, P. Extrinsic stochastic factors (solute partition) in gene expression inside lipid vesicles and lipid-stabilized water-in-oil droplets: a review. *Synth. Biol.* **3**, 1–16 (2018).
6. Blanken, D., van Nies, P. & Danelon, C. Quantitative imaging of gene-expressing liposomes reveals rare favorable phenotypes. *Phys. Biol.* **16**, 045002 (2019).
7. Nishimura, K. *et al.* Identification of giant unilamellar vesicles with permeability to small charged molecules. *RSC Adv.* **4**, 35224 (2014).
