# Supplementary material for: Intercellular communication between artificial cells by allosteric amplification of a molecular signal
Source: Nat Commun. 2020 Apr 3;11:1652. doi: 10.1038/s41467-020-15482-8 (PMC7125153; doi:10.1038/s41467-020-15482-8)
Supplement: Supplementary file 3 — Description of Additional Supplementary Files [file 41467_2020_15482_MOESM3_ESM.docx]

**Description of Additional Supplementary Files**

**Supplementary Movie 1: Sender GUVs generated a signalling front that activated the Receivers over long distances**

Time-lapse recording of a signalling front produced by Senders that activated Receivers in a distance-dependent fashion. Sender GUVs = green, Receiver GUVs = red, NADH = cyan. Scale bar = 50 µm.

**Supplementary Movie 2: Activation of Receivers by Senders, zoom**

Zoom of a region of Supplementary Movie 1. Scale bar = 50 µm.

**Supplementary Movie 3: Activation of Receptive Receivers only**

Time-lapse recording of the selective activation of Receptive Receivers (red) by Senders (blue). Unsusceptible Receivers (green) were not activated by the Senders. NADH = cyan. Scale bar = 50 µm. The membrane fluorophores in the GUVs (especially for the Senders) bleached during the recording, but the GUVs did not move or burst significantly.

**Supplementary Movie 4: Activation of Receivers by AMP**

Time-lapse recording of the activation of Receivers by 5 mM AMP and 1 mM Na_2_HPO_4_. Receiver GUVs = red, NADH = cyan. Note that the membrane fluorophore bleached somewhat during the recording, yet this did not affect the GUVs.

**Supplementary Movie 5: αHL insertion followed by calcein leakage from GUVs**

Time-lapse recording of calcein release from GUVs upon αHL insertion. GUV membrane composition was 70/30 DOPC/cholesterol + 0.06% DOPE-LRB (red). Calcein (1.0 mM, green) was loaded inside. [αHL] was 33 μg ml^–1^.
